# Post-operative rehabilitation in a traumatic rare radial nerve palsy managed with tendon transfers: a case report

**DOI:** 10.11604/pamj.2020.36.141.23994

**Published:** 2020-06-30

**Authors:** Pratik Phansopkar, Vrushali Athawale, Aachal Birelliwar, Waqar Naqvi, Swapna Kamble

**Affiliations:** 1 Department of Musculoskeletal Physiotherapy, Ravi Nair Physiotherapy College, Datta Meghe Institute of Medical Sciences, Wardha, Maharashtra, India,; 2 Ravi Nair Physiotherapy College, Datta Meghe Institute of Medical Sciences, Wardha, Maharashtra, India

**Keywords:** Rehabilitation, radial nerve palsy, tendon transfer, physiotherapy

## Abstract

Radial nerve is a frequently injured nerve. Radial nerve palsy result from direct trauma, neuropathies, and fracture over the humerus, malignant tumor and neuritis. A case of 26-year male is presented in this report who had a road traffic accident resulting in injury over the right shoulder, wrist joint and diagnosed of radial nerve palsy, consequently was operated with soft tissue reconstruction with tendon transfers which resulted into pain over wrist joint and loss of extensors muscle function of the wrist joint, which led to difficulty in performing activities of daily living. Surgical history and rehabilitation is mentioned in the case report. We report that there were significant improvements in muscle strength, range of motion, relief from pain, and exceptional improvements in the patient´s functional independence with physiotherapy interventions post-operative tendon transfers.

## Introduction

The radial nerve at the upper extremity is the most often injured nerve. Paralysis of the radial nerve is divided into open and close injury. Radial nerve palsy results from direct trauma, neuropathies, fracture over the humerus, malignant tumor, and neuritis. Radial nerve palsy treatment depends upon the level, duration, severity, and cause of injury. Radial nerve injury is treated conservatively and surgically by nerve repair, tendon transfer, and nerve grafting [1]. When nerve repair is impossible to restore the functional loss tendon transfer is done [2]. Radial nerve can be damaged at any site along its course. Radial nerve palsy is characterized by pain and paralysis of the extensor muscles of the wrist and fingers [3]. Physical therapy for radial nerve palsy following the transfer of tendons is described in this report. The tendon of palmaris longus was transferred to the tendon of extensor pollicis longus and tendon of flexor carpi radialis was transferred to the tendon of extensor digitorum communis.

## Patient and observation

**Figure 1:**
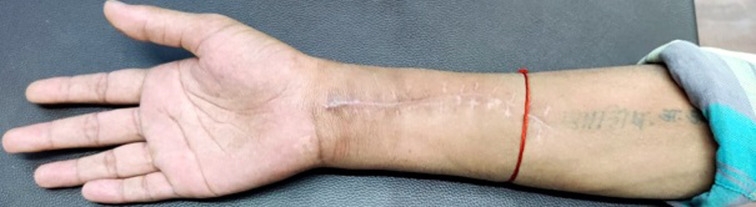
supinated forearm showing scar marks over distal forearm

**Figure 2:**
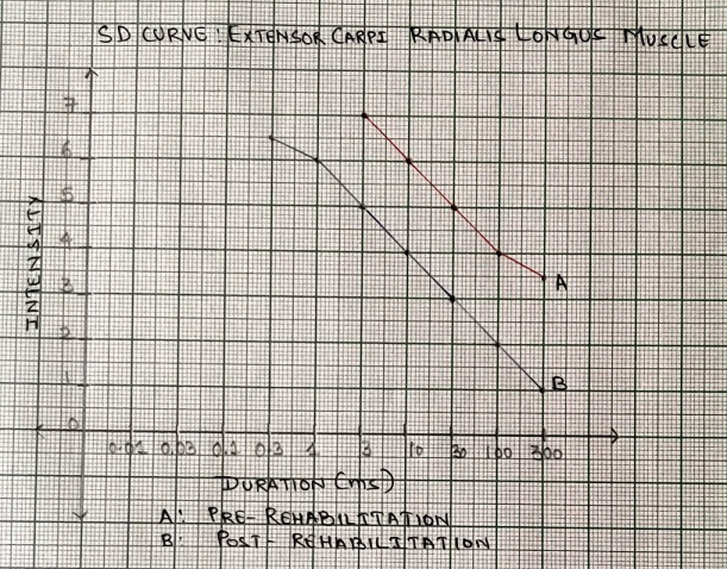
graph showing the strength-duration curve of extensor carpi radialis longus muscle pre and post rehabilitation; nature of curve (A: pre-rehab) is steep and cannot contract to impulse of shorter duration than 3 milliseconds; nature of curve (B: post-rehab) is less steep as compared to pre-rehab which suggests improvements in innervation

**Figure 3:**
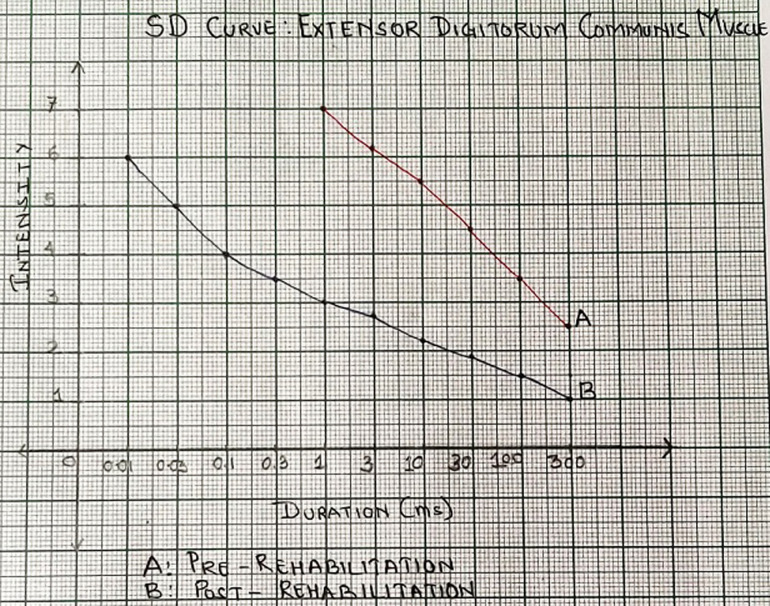
graph showing the strength-duration curve of extensor digitorum communis muscle; nature of curve (A: pre-rehab) is steep and cannot contract to impulse of shorter duration than 2 milliseconds; nature of curve (B: post-rehab) is less steep as compared to pre-rehab which suggests improvements in innervation and contracts to an impulse of 1 milliseconds

A 26 years old male, a barber by occupation with right-hand dominance met with a road traffic accident which resulted in injury around the right upper extremity, no history of head trauma and patient was conscious oriented and taken to Acharya Vinoba Bhave Rural Hospital (AVBRH), Sawangi Meghe. Orthopedic surgeon advised investigations X-ray, magnetic resonance imaging (MRI), and nerve conduction velocity (NCV) studies and was diagnosed with right axonotmesis of radial nerve (Grade 3 Sunderland´s classification) with no fracture involvement and was given static cock-up splint to stabilize the wrist and hand and analgesic medications were prescribed and advised follow-up after 15 days. On follow-up, there were no improvements in strength and symptoms and hence was posted for soft tissue reconstruction of the right upper limb with a tendon transfer. The tendon of flexor carpi radialis was harvested and attached to tendon of extensor digitorum communis and tendon of palmaris longus was harvested and attached to tendon of extensor pollicis longus. After the surgery, the right forearm was placed in a plaster cast for 3 weeks. On removal of plaster cast, he visited for rehabilitation in the physical therapy department with the complaints of pain over the wrist joint and difficulty in holding objects between thumb and fingers of the right hand. Character of pain was dull aching; tingling over the distal forearm, along with numbness and paraesthesia in the palm (numerical pain rating scale: 6/10). Scar was present over the dorsal and volar aspect of the distal forearm (Figure 1).The range of motion has been depicted in Table 1. Elbow, wrist, thumb muscle strength examination according to medical research council was carried out (1/5: wrist and thumb extensors, 3/5: elbow supinators and pronators, wrist and thumb flexors, 4/5: elbow flexors and extensors). All superficial and deep sensations were intact. Functional outcome by patient-specific functional scale revealead score 2/10 strength duration curve revealed the status of denervation (Figure 2, Figure 3). Four weeks of rehabilitation was carried out with each week adding a different component to the treatment. Rehabilitation consisted of faradism under pressure with a surged duration of 1 second and surge interval of 3 seconds with intensity enough to produce visible contractions with passive movement of wrist joint for 10 repetitions, scar tissue immobilization as an adjunct to stretching. The duration of treatment was 2 hours; this protocol was followed for one week. The patient was advised for dynamic cock-up splint for maintaining the position of joints. Ultrasound with 3 MHZ for 7 minutes followed by hot fomentation and wrist joint mobilization was executed for an increasing range of motion. Stimulation to wrist and finger extensors with intermediate galvanic current passive movements were incorporated to improve the muscle re-education. This protocol was carried out for one week. On the 1^st^ day of rehab along with electrical muscle stimulation (EMS) and joint mobilization, resistance training was initiated for wrist flexors and extensors, forearm supinator, and pronators with the help of resistance devices with 3 seconds hold for 15 repetitions. Different designed objects were used to improve the grip function. On the 21^st^ day of rehab, EMS parameters were switched to faradic current for reeducation. Strengthening exercises were continued and progression by increasing the intensity and duration based on clinical improvements. Post rehabilitation on 28^th^ day there were significant improvements in parameters of pain intensity (NPRS 2/10), muscle strength (3+/5), range of motion (Table 1), patient-specific functional scale score (7/10) and strength-duration curve showed the status of innervations (Figure 2, Figure 3).

**Table 1:** range of motion assessment on 1^st^ day and 28^th^ day of rehabilitation

	1^st^ day of rehabilitation		Post-rehabilitation 28^th^ day	
	Active rom	Passive rom	Active rom	Passive rom
Wrist flexion	0-600	0-650	0-700	0-750
Wrist extension	Unable to perform	0-200	0-100	0-200
Thumb extension	Unable to perform	0-10	0-20°	0-25°
Ulnar deviation	0-250	0-250	0-300	0-350
Radial deviation	Unable to perform	0-100	0-20°	0-200
Forearm pronation	0-600	0-700	0-700	0-750
Forearm supination	0-700	0-800	0-800	0-850

## Discussion

Forearm tendon transfers is an acceptable treatment method for irreparable radial nerve injuries [4]. In our case report patient´s main concerns were pain over wrist joint, difficulty in grasping the objects, and decrease in range of motion post tendon transfer surgery. Splinting prevents from over-stretching of the denervated group of muscles, prevents from joint contractures, and preserves the movements [5]. Cock-up splint served to the purpose of maintaining the position of the wrist and hand in our patient. Thermotherapy was advised in form of application of hot pack which resulted in an increase in body temperature, blood flow, extensibility of soft tissues which helped in the healing process by removal of waste debris, relief from pain, and thereby relaxing the muscles [6]. Ultrasound plays an important role in inhibiting the sympathetic activities, enhance the viscoelastic properties, decreases the stiffness of joint in combination with joint mobilization to achieve significant improvements in restoring range of motion as discussed by David *et al*. [7]. In our patient ultrasound application helped to inhibit the sympathetic activity by reducing pain post sessions. There are various retrospective, prospective, systemic reviews and case reports on surgical management (tendon transfers and nerve transfers) of radial nerve injuries, but there is paucity towards a structured physical rehabilitation following this surgical management [4,8,9]. Electrical muscle stimulation was incorporated for denervated muscles, initially intermediate galvanic current to stimulate the muscles followed by the faradic current for muscle reeducation. EMS helps to increase the range motion, improve muscle performance, and prevent contractures which were reported by Powell *et al*. [10]. Masoud *et al*. reported improvements in patient concerns of pain and mobility following tendon transfer surgery for radial nerve palsy patients which were not significant, however, rehabilitation was initiated immediately after surgery [11]. In this case, strengthening exercises with manual and mechanical resistance device progressively helped to increase the muscle strengths, and thereby making the patient functionality independent. Elizabeth *et al*. discussed the importance of initiating physical therapy post tendon transfer surgery for radial nerve damage after 4 weeks, nevertheless, in our study, the post-operative rehabilitation after 3 weeks of splint removal led to improvements in the patient´s function [12]. In our case, an adjunct of therapeutic interventions aided in successful improvements in patient´s functional outcomes illustrating the need to target the specific limitations and importance of physiotherapy post tendon transfer surgeries in radial nerve injuries.

## Conclusion

Radial nerve injuries can be limiting physical functions lifelong. The present case report is exceptional rare after tendon transfers. Following four weeks of rehabilitation in such a case, there was significant improvement in muscle strength and functional independence through strengthening exercises, muscle reeducation, electrotherapy, and other physical therapy techniques. This case report emphasizes to embellish the necessity of a structured physical rehabilitation and its importance following tendon transfer surgeries in radial nerve injuries to attain a successful recovery to the patient.
